# Transrectal drainage of a diverticular abscess using a pigtail catheter without radiological guidance: a case report

**DOI:** 10.1186/1752-1947-5-1

**Published:** 2011-01-04

**Authors:** Bobby VM Dasari, John Lawson, Jack Lee

**Affiliations:** 1 Level 2, Department of General Surgery, Belfast City Hospital, Belfast, BT9 7AB, UK; 2 Surgical Assessment Unit, Level 3, Daisy Hill Hospital, Newry, County Down BT35 8DR, Northern Ireland, UK; 3 Department of Radiology, Belfast City Hospital, Belfast, BT9 7AB, UK

## Abstract

**Introduction:**

Percutaneous or endocavitory drainage of a diverticular abscess under radiological guidance often enables one to perform a one-staged resection and anastomosis (without stoma formation) instead of a two-staged procedure. It reduces the significant postoperative morbidity and mortality associated with the conventional emergency surgical management. However, radiological guidance is not always available due to limited resources during out-of-hours.

**Case presentation:**

A 78-year-old Caucasian woman underwent transrectal drainage of a diverticular abscess performed with a pigtail catheter without radiological guidance. Technical details of the procedure are described and alternative options discussed.

**Conclusion:**

In carefully selected patients, per-rectal drainage using a pigtail catheter can be performed without radiological guidance and the procedure offers a simple and effective way of controlling sepsis.

## Introduction

Diverticular abscess is the most common complication of acute diverticulitis [1]. Patients with diverticular abscess were historically managed by a three-staged procedure - drainage of abscess with diversion colostomy followed by resection of diseased segment of the bowel and, finally, restoration of bowel continuity. Hartmann's procedure, with a second operation for closure of colostomy, emerged in 1980s as an effective alternative to the three-staged procedure. However, it is associated with significant mortality (4%-10%) and morbidity (up to 40%) [2]. Keck and colleagues reported mortality in 2% and an anastomotic leak rate in 4% of patients who underwent reversal of the Hartmann procedure [3].

Primary resection and anastomosis, with or without a diversion, is advocated in the management of complicated diverticular disease with reported mortality rates of 6%-9% [4]. However, the appropriate operative option is often influenced by pre-operative prognostic factors such as age, co-morbidities, duration of symptoms, clinical presentation of the patient, intra-operative findings and the level of experience of the surgeon. In general, the more diffuse and severe the peritoneal soiling and contamination, the less one is inclined to perform a primary anastomosis [5].

After the development of computed tomography (CT) guided percutaneous transabdominal drainage of intra abdominal abscesses (in 1980s), the procedure was widely adopted for the management of pericolic diverticular abscesses. Large localized abscesses (> 4-5 cm) are primarily drained by a percutaneous approach in order to resolve the sepsis. Resuscitation with intravenous fluids, antibiotics and adequate analgesia remains an integral part of the management. This is followed by elective single-staged resection and anastomosis of the sigmoid colon once the acute inflammation in the colon and pericolic tissue subsides. Percutaneous drainage of an abscess is successful in allowing a later, more elective, single-stage resection and anastomosis in 74% of patients with diverticular abscess [6].

Those with smaller abscesses are often treated with intravenous antibiotic therapy alone [1]. Percutaneous abscess drainage is a safe and effective alternative to surgery for draining infected fluid collections, with a higher success rate (70%-90%) [7], lower complication rate and shorter hospital stay compared to surgical drainage [8]. However, 20%-25% of patients are either not suitable for radiological drainage (multiloculated, anatomically inaccessible) or do not respond to drainage and will require surgical intervention [9].

Deep pelvic abscesses are not always accessible for trans-abdominal percutaneous drainage and are managed by transvaginal or transrectal drainage under radiological (ultrasound/CT/fluoroscopy) or endoscopic guidance. We describe a case of complicated diverticular disease associated with a pelvic abscess successfully managed by transrectal drainage with a pigtail catheter without image guidance.

## Case presentation

A 78-year-old Caucasian woman was admitted with lower abdominal pain, diarrhea associated with fever, chills and rigors of three weeks duration. Her past medical history included cerebral vascular accidents, atrial fibrillation, bronchial asthma and hypothyroidism. Relevant drug history included digoxin, aspirin and clopidogrel. She received oral co-amoxiclav (amoxicillin trihydrate 500 mg + clavulanic acid 125 mg three times a day) for five days in the community. On examination, she was pyrexic (38°C) with tachycardia (fast atrial fibrillation: 128/min) and tachypnea (respiratory rate: 22/min). She was morbidly obese with a Body Mass Index of 41 Kg/m^2^. Abdominal examination showed tenderness in the left iliac fossa. There was no localized peritonism or a palpable mass.

**Figure 1:**
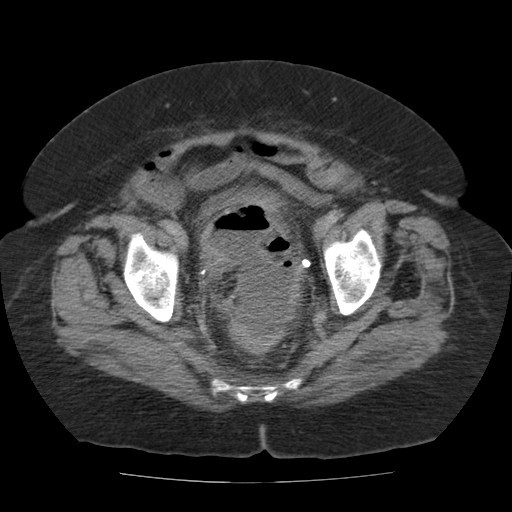
**Computed tomography scan of the abdomen and pelvis (axial section) demonstrating the diverticular abscess extending anterior to rectum**.

Blood investigations showed raised inflammatory markers [white cell count (WCC): 24.6 × 10^9^/L and C-reactive protein: 253/L]. A CT scan of the abdomen and pelvis revealed a large pelvic abscess measuring 12.5 cm × 6.3 cm (Figure 1) associated with sigmoid diverticular disease. The abscess was considered for transrectal drainage as the pelvic collection was not suitable for safe percutaneous transabdominal drainage under CT guidance due to an overlying sigmoid colon. There was no on-call interventional radiologist available to drain the abscess under radiological guidance. Therefore, following detailed assessment of the anatomical location of the abscess, transrectal drainage was performed using a pigtail catheter without radiological guidance.

The patient was taken to theater after adequate resuscitation with intravenous fluids and intravenous antibiotics (piperacillin and tazobactam 4.5 gm and metronidazole 500 mg three times a day). She was placed in the lithotomy position under general anesthetic and a per-rectal examination was performed. The abscess was abutting the anterior wall of the mid rectum on digital examination. A 22G epidural needle attached to a 10 mL syringe was inserted into the pelvic abscess through the rectum. Aspiration of pus confirmed the position of needle. The epidural needle was left in place to mark the direction and depth of the abscess cavity. A 10 Fr Hydrophilic - Coated Nephro (UreSil, IL, USA) catheter with 27 cm working length and a locking pigtail was inserted into the abscess cavity just adjacent to the guiding epidural needle and 200 mL of pus was drained. The abscess cavity was irrigated with 0.9% saline until the aspirates appeared clear. The catheter was secured in place by locking the pigtail.

**Figure 2:**
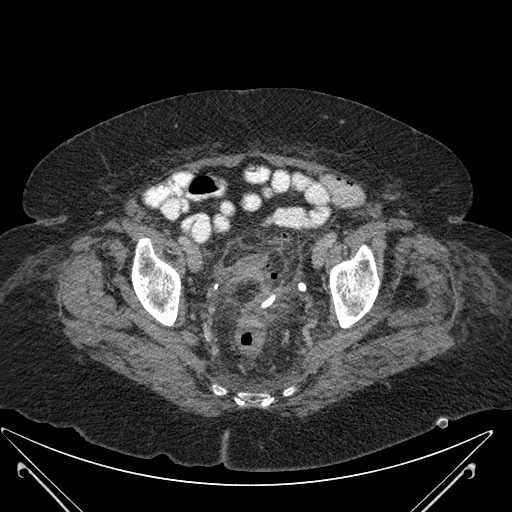
**Computed tomography scan of the abdomen and pelvis (axial section) after drainage of the abscess with catheter *in-situ***.

**Figure 3:**
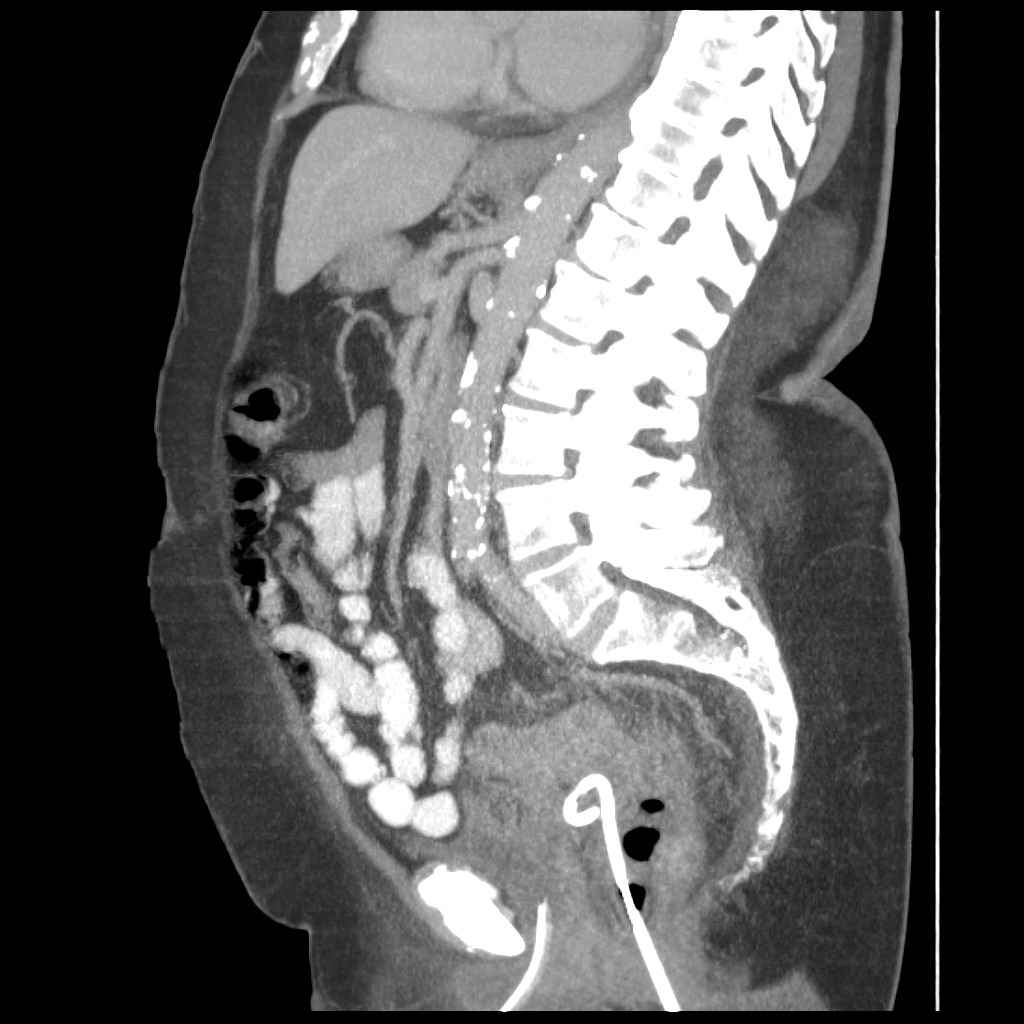
**Computed tomography scan of the abdomen and pelvis (sagital section) demonstrating the transrectal placement of a pigtail catheter**.

There was significant improvement in the clinical condition of the patient and the inflammatory markers began to reduce the day after procedure (WCC: 7.3 × 10^9^/L, C-reactive protein: 121 mg/L) and returned to normal within the next 72 h. A drainage catheter was flushed with 30 mL of normal saline twice a day. A repeat CT scan of abdomen and pelvis, performed on day four after the procedure, revealed mild pericolic inflammatory changes in the sigmoid colon. The catheter was within the abscess cavity with no significant residual pelvic collection (Figures 2 and 3). It spontaneously dislodged on day five after the procedure. Intravenous antibiotics were continued for nine days and the patient was discharged home 10 days after the procedure. A flexible sigmoidoscopy and barium enema examination performed six weeks later confirmed sigmoid diverticular disease. Due to the considerable risk associated with this patient's medical co-morbidities, an elective sigmoid colectomy was not performed. She remained symptom-free six months later.

## Discussion

Primary drainage of a diverticular abscess via the percutaneous transabdominal route reduces the mortality and morbidity associated with emergency open surgery. However, deep pelvic abscesses are not always amenable for transabdominal drainage as the pelvic bones, intestine, bladder, iliac vessels and gynecologic organs may preclude safe access. Percutaneous transgluteal or endocavitary (transvaginal or transrectal) approaches are the alternative methods for obtaining safe access to deep pelvic abscesses.

The transgluteal approach provides the best access to presacral abscess and catheter fixation is more easily achievable compared to endocavitory approaches. Injury to the superior or inferior gluteal vessels, resulting in significant bleeding and injury to the sciatic nerve, is a potential complication, the risk of which can be reduced by a CT-guided infra piriformis approach. Discomfort to the patient and kinking of catheter when lying in supine position are the drawbacks of placing the catheter through the gluteal region [10].

The endocavitory approach (transvaginal and transrectal) is safer as fewer vital structures are at risk of injury and the patient acceptance is greater than transgluteal placement of the catheter. Transvaginal drainage performed in women is technically more difficult, more painful and is a lengthy procedure than the transrectal approach due to the inherent thickness of the vaginal wall muscles [11]. Furthermore, the transvaginal route is not suitable for drainage of presacral collections due to the interposition of rectum. Iatrogenic colovaginal fistula may complicate abscess drainage associated with Crohn's disease but this complication is less common with primary diverticular pathology.

Drainage by the transrectal route offers a quick and safe access into pelvic abscess via short and direct route. The additional image guidance provides safe access to the clinically impalpable abscesses. Transrectal catheter drainage under ultrasound, fluoroscopy, CT or endoscopic guidance is more popular than the traditional transrectal incision and drainage. Fecal contamination of the abscess cavity by transrectal drainage of an abscess is not a concern as raised intra abdominal pressures during defecation empty the abscess cavity preferentially [12]. This case report suggests that, in selected patients, the traditional transrectal drainage performed without radiological guidance still has a role to play in the management of pelvic abscesses.

There is no published study comparing the different techniques because the procedure of choice depends on the site of abscess cavity, availability of ultrasound/CT scans and, finally, the preference and experience of the radiologist or surgeon. Nonetheless, success rates of 85%-100% are reported in most of the series which have evaluated individual techniques in the management of intra abdominal and pelvic abscesses [10,13,14].

Radiological guidance is not always available due to limited resources, especially out-of-hours. It is not uncommon to come across an on-call radiologist who does not perform interventional procedures. In such circumstances, catheter drainage of the abscess may be performed without image guidance. However, it is important that the surgeon undertakes a detailed study of the radiological and clinical findings before opting to undertake such a procedure. Abscesses that are extending deep into the pelvis and abutting the mid rectum are more suitable for this procedure.

Our patient was toxic with significant co-morbidities and was at high risk of post surgical mortality and morbidity. The deep pelvic abscess was not particularly suitable for percutaneous drainage and there was no on-call interventional radiologist available to drain it. With the favorable location of the abscess, transrectal drainage was successfully performed without any radiological guidance using a pigtail catheter.

Patency of the drainage catheter is maintained by flushing with normal saline two to three times a day. This prevents the tube clogging with debris that can cause an apparent reduction in the catheter output. The drainage catheter should be removed when the output from the drain decreases and there are improved constitutional symptoms, clinical signs and inflammatory markers. If the clinical findings are equivocal, radiological imaging helps to reassess the size of abscess cavity and to confirm the position of catheter. The catheter may need repositioning if dislodgement occurs. Spontaneous expulsion of a catheter is common and this does not necessarily compromise the success of the procedure as rapid evacuation and collapse of the cavity occur due to intra-abdominal pressure and dependent drainage.

## Conclusion

In carefully selected patients with deep pelvic abscess, transrectal drainage using a pigtail catheter can be performed without radiological guidance and the procedure offers a simple and effective way of controlling sepsis.

## Competing interests

The authors declare that they have no competing interests.

## Consent

Written informed consent was obtained from the patient for publication of this case report and any accompanying images. A copy of the written consent is available for review from the journal's Editor-in-Chief.

## Authors' contributions

All authors have read and approved the final manuscript. BVD was involved in the conception of the report, literature review, manuscript preparation, editing and submission. JLa was involved in the manuscript editing and review. JLe was involved in the manuscript editing and review.
